# Volitional exaggeration of body size through fundamental and formant frequency modulation in humans

**DOI:** 10.1038/srep34389

**Published:** 2016-09-30

**Authors:** Katarzyna Pisanski, Emanuel C. Mora, Annette Pisanski, David Reby, Piotr Sorokowski, Tomasz Frackowiak, David R. Feinberg

**Affiliations:** 1 Department of Psychology, Neuroscience & Behaviour, McMaster University, Canada; 2 Institute of Psychology, University of Wrocław, Poland; 3 Mammal Vocal Communication & Cognition Research Group, School of Psychology, University of Sussex, United Kingdom; 4 Department of Animal and Human Biology, Faculty of Biology, University of Havana, Cuba; 5 Instituto de Ciencias Biomedicas, Universidad Autonoma de Chile, El Llano Subercaseaux 2801, San Miguel, Santiago, Chile

## Abstract

Several mammalian species scale their voice fundamental frequency (*F*0) and formant frequencies in competitive and mating contexts, reducing vocal tract and laryngeal allometry thereby exaggerating apparent body size. Although humans’ rare capacity to volitionally modulate these same frequencies is thought to subserve articulated speech, the potential function of voice frequency modulation in human nonverbal communication remains largely unexplored. Here, the voices of 167 men and women from Canada, Cuba, and Poland were recorded in a baseline condition and while volitionally imitating a physically small and large body size. Modulation of *F*0, formant spacing (∆*F*), and apparent vocal tract length (VTL) were measured using Praat. Our results indicate that men and women spontaneously and systemically increased VTL and decreased *F*0 to imitate a large body size, and reduced VTL and increased *F*0 to imitate small size. These voice modulations did not differ substantially across cultures, indicating potentially universal sound-size correspondences or anatomical and biomechanical constraints on voice modulation. In each culture, men generally modulated their voices (particularly formants) more than did women. This latter finding could help to explain sexual dimorphism in *F*0 and formants that is currently unaccounted for by sexual dimorphism in human vocal anatomy and body size.

Several mammalian species are known to scale their vocal frequencies (see refs 1, 2, 3, 4 for reviews). Red deer (*Cervus elaphus*) offer a prime example, wherein stags drastically lower their larynges to extend their vocal tracts during roaring and do so predominantly in response to threatening male competitors^5^,^6^. This behaviour lowers formants beyond what would be expected based on mammalian acoustic allometry, thus exaggerating the animal’s apparent size. Several researchers have proposed similar capabilities in humans, suggesting that systematic voice frequency modulation for size exaggeration should be observed not only across mammalian species^3^, but also across human cultures^7^,^8^. Others have further hypothesized that exaggeration of body size through voice frequency modulation may have contributed to the descent of the human larynx^9^, and is likely to have played a critical role in the early evolution of nonverbal communication, ultimately paving the way for the emergence of articulated speech^4^,^9^. The present study is the first to empirically test whether men or women do in fact systematically modulate *F*0 and formants when instructed to deliberately alter their apparent body size.

## Anatomical constraints on voice frequencies

Guided by the source-filter theory of speech production^10^,^11^, behavioral scientists studying acoustic communication of body size in humans and other mammals have focused on two voice features: fundamental frequency (*F*0) and vocal tract resonances (formants). Voice *F*0 is produced by the vocal folds, whose rate of vibration is related to their mass, length and tension, whereas the supralaryngeal vocal tract filters the voice producing formants that are inversely related to supralaryngeal vocal tract length^12^. Voice *F*0 and formants affect our perception of pitch and timbre, respectively, and play a major role in speech articulation^13^. These voice features are also highly sexually dimorphic and have likely undergone intense sexual selection in humans^14^.

Formants scale fairly allometrically with vocal tract length and body size^15^, because the mammalian vocal tract is constrained by the skeletal structures that surround it. In contrast, although larger vocal folds produce a lower *F*0, the larynx grows largely independently of the rest of the body and *F*0 does not therefore scale allometrically with body size in humans^16^. Indeed, formants explain several times more variation in body size than does *F*0 when sex and age are controlled^17^. Nevertheless, among humans, neither vocal feature explains a substantial portion of the variance in body size at the within-sex level^17^,^18^.

The lack of a robust physical relationship between the human voice and body size suggests a lack of constraints to maintain allometry. Volitional voice modulation to exaggerate body size should therefore be possible, and could help to further explain this puzzling disassociation. At the perceptual level, and despite the lack of robust physical relationships, listeners cross-culturally associate both low *F*0 and low formants with large body size even within sexes^19^,^20^,^21^,^22^,^23^. This further suggests that similar to other mammals (see e.g. refs 24 and 25) the human voice conveys both honest and exaggerated cues to size. Perceptual correspondences between low voice frequencies and large body size are important because they may drive selection for vocal communication (or exaggeration) of size, even in the absence of robust physical relationships between the voice and body.

## Morphological modifications for size exaggeration

The vocal anatomy of many mammals has undergone morphological modifications that appear to function, at least in part, to exaggerate apparent size^1^. These include non-laryngeal velar vocal folds in koalas (*Phascolarctos cinereus*) that allow males to produce *F*0’s typical of an animal as large as an elephant^26^, the subhyoid air sacs in black-and-white colubus monkeys (*Colobus guereza*) that amplify resonant frequencies^24^, and the descended larynx in males of several polygynous deer species^6^, and koalas^27^, that enable them to produce low formant frequencies characteristic of much larger species.

Humans also have a descended larynx. In humans the descended larynx allows for the production of a broader range of speech sounds relative to the vocal repertoires of other primates^28^, but importantly, also results in a lengthened pharyngeal cavity and thus relatively lower formants^9^. Among men, pubertal hormones cause the larynx to descend even further, a full vertebra lower than among women^16^, and cause men’s vocal folds to grow 60% larger than women’s^29^. These morphological modifications are evolutionarily relevant, as they implicate a role of sexual selection and size exaggeration in the evolution of human vocal frequencies. However, men’s *F*0 and formants are approximately 80% and 20% lower than women’s, respectively, and these sex differences in *F*0 and formants exceed that which can be explained by sexual dimorphism in the vocal anatomy (i.e., men’s vocal folds are on average only 60% larger than women’s, and their vocal tracts are typically 15% longer) or by sexual dimorphism in body size (men are on average only 10% taller than women)^30^. This discrepancy alludes to possible behavioural differences between men and women in vocal production or modulation^31^, wherein men may lower their *F*0 and formants more than women through the behavioural mechanism of voice modulation. If true, voice modulation may account for some portion of the unexplained variance between men and women’s vocal frequencies.

## Voice frequency modulation in humans

Mechanistically, volitional modulation of *F*0 is achieved by manipulating the tension and effective length or surface area of the vocal folds using the laryngeal muscles (cricothyroid muscles lengthen the vocal folds and increase *F*0, whereas thyroarytenoid muscles shorten the vocal folds and decrease *F*0, and their opposing effects can be coordinated or independent)^32^,^33^ or by increasing subglottal pressure. In contrast, lowering the larynx or protruding the lips increases supralaryngeal vocal tract length and reduces formant spacing^13^,^32^,^33^. Although recent investigations suggest some flexible control of voice frequencies in nonhuman primates^34^,^35^,^36^, the ability to intentionally and volitionally modulate source and filter components is uniquely advanced in humans and is thought to constitute a precursor of speech^4^,^9^. Indeed volitional voice modulation in humans involves comparatively complex neural processes that are absent in other mammals, including nonhuman primates^37^.

Infant directed speech, in which adults speak with higher *F*0 and exaggerated prosodic cues when addressing infants compared to older individuals, represents perhaps the most extensively studied form of voice modulation in humans and appears to be present across diverse cultures^38^,^39^. More recently, a small number of empirical studies have begun to examine voice modulation as a social tool used to exploit ecologically relevant traits, and among these, almost all have focused on *F*0 modulation (see ref. 4 for review). For example, in a series of recent studies, Cartei and colleagues^40^,^41^,^42^ showed that men, women, and children volitionally decreased both *F*0 and formants when asked to sound masculine, and increased both voice features to sound feminine. Several studies report *F*0 modulation in men or women when speaking to a potential mate^43^,^44^,^45^,^46^,^47^,^48^ or competitor^49^. In the context of mate preferences, these studies have found that both sexes volitionally modulate *F*0 when instructed to speak in a more attractive voice^43^ as well as when directing their speech toward an attractive person of the opposite sex^45^,^47^.

Voice modulation may therefore be utilized to deemphasize or accentuate various indexical traits and this may be evolutionary adaptive. In particular, men who can effectively exaggerate their apparent body size through *F*0 and formant modulation may reap the social benefits associated with physical largeness, such as increased access to resources and mates. Indeed, taller men, and those with relatively lower voice *F*0 and formants indicating larger body size, are typically preferred as mates by women across a diverse range of cultures^50^. Nevertheless, to be effective, vocal modulation of body size should exceed the just-noticeable differences in *F*0/formant perception^23^,^51^,^52^ and should have the intended effects on listeners’ social assessments. While some studies have found that volitional voice modulation effectively increased listeners’ assessments of the vocalizer’s attractiveness, competence, and intelligence^43^,^47^, one study found that sex-typical *F*0 modulation influenced listeners’ assessments of dominance but not voice attractiveness^46^.

## The Present Study

The present study is the first to test whether humans can modulate voice features known to be associated with body size (fundamental and formant frequencies) when instructed to deliberately alter their apparent body size. In addition, we examined whether this voice modulation reflects real (physical) and perceived relationships between the human voice and body (i.e., lower *F*0 and formants indicate larger size and *visa versa*), whether the behaviour differs between the sexes, and whether the behaviour is present cross-culturally.

We tested these hypotheses in 167 men and women from three distinct cultures and language groups: Canada (English), Cuba (Spanish), and Poland (Polish). Participants were recorded speaking vowel sounds in their baseline voice and while imitating a physically large and small body size. We predicted that participants would lower *F*0 and formants (increase apparent vocal tract length, VTL) to convey large size, and raise voice *F*0 and formants (reduce VTL) to convey small size. We further predicted that men would modulate their voices more than women, thereby accounting for some of the unexplained sexual dimorphism in *F*0 and formants. In contrast, we predicted that patterns of voice modulation would not differ across the three cultures. This latter finding would provide some support for fairly universal sound-size correspondences, and/or anatomical or biomechanical constraints on voice modulation.

The present study was specifically designed to test for the first time whether adult speakers are capable of volitional adjustments to their larynx (fundamental frequency modulation) and vocal tract (formant frequency modulation) in a manner that parallels the known relationships between these vocal parameters and body size in humans. Acoustic analyses were utilized to measure voice frequency parameters and to test whether these modulations exceed just-noticeable differences in *F*0 and formant perception. However in the present study we did not test whether these modulations effectively alter listeners’ perceptions of the vocalizer’s body size.

## Results

**Figure 1:**
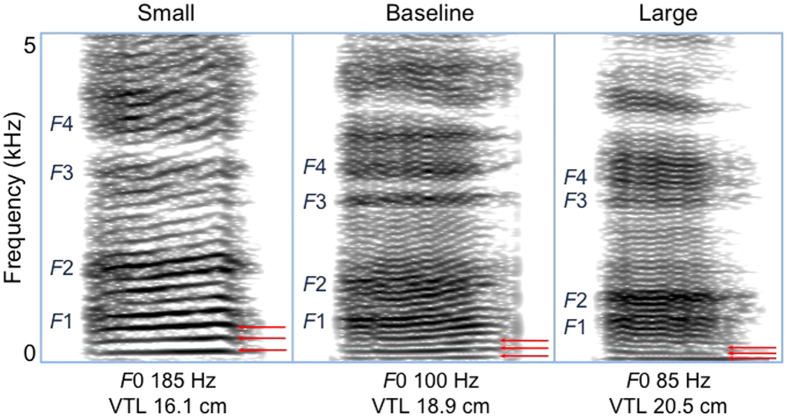
Spectrograms illustrating the vowel /a/ spoken by the same adult male in each condition. Formants (*F*1–*F*4) are labeled. Fundamental frequency and the first two harmonics (multiple integers of *F*0) are indicated by red arrows. Participants raised formants and F0 to sound small, thus increasing spacing between *F*1-*F*4 and between harmonics (left), and lowered formants and F0 to sound large (right). Gaussian FFT, window length 0.04; dynamic range 60 dB. Refer to Supplementary Audio S1 for corresponding voice recording.

Table 1 shows unstandardized means and maxima in VTL and *F*0 modulation for each sex and condition. As predicted, both sexes decreased VTL and increased *F*0 to sound small, and increased VTL and decreased *F*0 to sound large (Fig. 1; Supplementary Audio S1). Notably, men increased their apparent VTLs by as much as 25% to portray a physically larger body size, and increased their *F*0 by up to three times the baseline frequency (i.e., almost 300%) to sound smaller, reaching pitch registers characteristic of a child^53^.

### Formant or vocal tract length modulation

**Figure 2:**
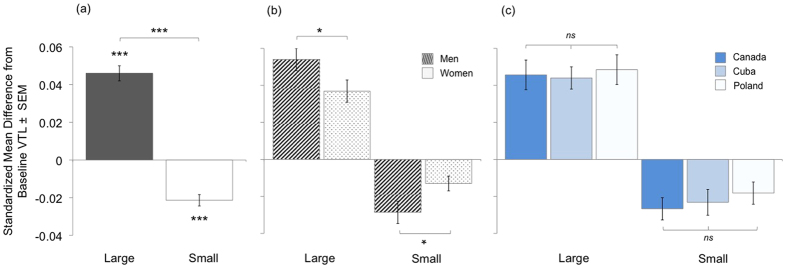
Vocal tract length (VTL) modulation given as the standardized difference from baseline in the large and small conditions. **(a)** Participants increased VTL to sound physically large and decreased VTL to sound small. The magnitude of VTL modulations was greater in the large than small condition. **(b)** Men modulated their VTLs more than did women in both size conditions. **(c)** VTL modulation did not vary cross-culturally. ***p < 0.001, *p < 0.05, *ns p* > 0.05.

An analysis of variance revealed a main effect of condition (large versus small body size imitation)(*F*_1,111_ = 109.2, *p*<0.001, 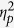 = 0.50; Fig. 2a) and an interaction between condition and sex (*F*_1,111_ = 8.1, *p* = 0.005, 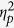 = 0.07; Fig. 2b) on VTL modulation. There were no other significant effects (all *F* < 2.1, all *p* *>* 0.13) including no effects of culture (Fig. 2c). Post-hoc analyses showed that participants increased their VTL from baseline in the large condition (one-sample *t*_132_ = 9.7, *p* < 0.001) and decreased their VTL in the small condition (*t*_132_ = −5.4, *p* < 0.001). Moreover, men increased VTL in the large condition (one-way *F*_1,132_ = 6.01, *p* = 0.016) and decreased VTL in the small condition (*F*_1,122_ = 5.78, *p* = 0.018) significantly more than did women. A model examining *absolute* differences from baseline (i.e., magnitude of modulation) indicated that VTL modulations were more extreme in the large than small condition, and more extreme among men than women in both conditions (see Supplementary Information; see also Fig. 2).

### Fundamental frequency modulation

**Figure 3:**
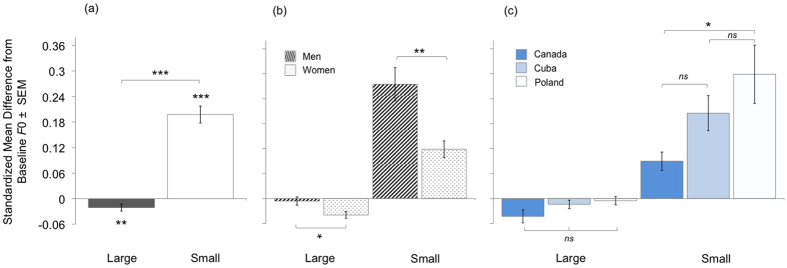
Fundamental frequency (*F*0) modulation. **(a)** Participants decreased *F*0 to sound physically large, and increased *F*0 to sound small. The magnitude of *F*0 modulations was greater in the small than large condition. **(b)** Men modulated VTL more than did women, but only in the small condition. **(c)** *F*0 modulation did not vary cross-culturally in the large condition, however in the small condition, Poles modulated their *F*0 more than did Canadians. ***p < 0.001, **p < 0.01, *p < 0.05, *ns p* > 0.05.

We observed main effects of condition (*F*_1,161_ = 55.77, *p* < 0.001, 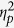 = 0.26; Fig 3a), sex (*F*_1,161_ = 10.7, *p* = 0.001, 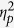 = 0.06; Fig 3b) and culture (*F*_2,161_ = 6.1, *p* = 0.003, 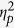 = 0.07; Fig 3c) on *F*0 modulation. These effects were qualified by a significant interaction between condition and sex (*F*_2,161_ = 4.4, *p* = 0.037, 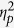 = 0.03) and a marginally non significant interaction between condition and culture (*F*_2,161_ = 3.1, *p* = 0.051, 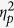 =0.04). There were no other significant effects (all *F* < 1.9, all *p* *>* 0.16).

Planned post-hoc analyses showed that participants decreased their *F*0 in the large condition (one-sample *t*_166_ = −2.6, *p* = 0.01) and increased their *F*0 in the small condition (*t*_166_ = 6.7, *p* < 0.001). Men increased their *F*0 more than did women to sound small (one-way *F*_1,166_ = 7.2, *p* = 0.008), however women decreased their *F*0 more than did men to sound large (*F*_1,166_ = 5.5, *p* = 0.021). Cultural differences in *F*0 modulation emerged only in the small condition (*F*_2,166_ = 4.4, *p* = 0.014), and only between Canadians and Poles (Fisher’s LSD *p* = 0.004; all other *p* > 0.11; Fig. 3c). A model examining absolute magnitude indicated that *F*0 modulations were more extreme in the large than small condition. Within the small condition, *F*0 modulations were more extreme among men than women (see Supplementary Information; see also Fig. 3).

## Discussion

The capacity for humans to volitionally modulate the source and filter components of our voices has traditionally been studied in the context of speech and language production^9^,^11^. The extent to which we modulate our voices for nonverbal communication, for instance to sound more masculine/feminine or attractive, has been investigated in comparatively few empirical studies^40^,^41^,^43^,^44^,^45^,^46^,^47^,^48^,^54^,^55^. Our study provides the first evidence that men and women from diverse cultures can spontaneously and volitionally modulate their fundamental and formant frequencies with the intent to exaggerate or reduce apparent body size, and that regardless of culture, men generally modulate their voices more than do women in this context. Acoustic analyses indicated that these modulations were in the predicted direction, such that men and women lowered *F*0 and formants when instructed to sound large, and increased *F*0 and formants when instructed to sound small, and that in most cases these modulations exceeded the just-noticeable differences in *F*0 and formant perception.

The patterns of voice frequency modulation observed in our study map onto real physical relationships between the voice and body, as larger people generally have lower formants and *F*0 than do smaller people^17^,^18^,^22^. However, because neither vocal parameter (especially *F*0) can explain a substantial proportion of the variance in human body size when sex and age are controlled^17^,^18^,^22^, volitional voice modulation of these parameters may also reflect an exploitation of listeners’ perceptual biases linking low voice frequencies to large body size and dominance^7^,^8^,^21^,^22^,^23^,^54^, or more general sound symbolic correspondences^56^. Indeed our results support Ohala’s prediction that similar voice frequency modulations will be observed across cultures, reflecting a universal “frequency code”^7^,^8^. It has also previously been suggested that perceptual biases based on the laws of physics, such that large objects resonate at lower frequencies, are likely to be cross-culturally universal precisely because they are determined by physics, not culture^57^ (see also ref. 3). Our cross-cultural results may alternatively reflect constraints on voice production in humans. Formants are especially constrained by the bony anatomy surrounding the vocal tract^15^, which is likely to impose upper and lower limits on formant modulation.

The sex differences in voice modulation observed here may be tied to a number of factors, most parsimoniously to differences in the vocal anatomy of men and women. For example, a longer supralaryngeal vocal tract among men may allow for greater laryngeal mobility that could result in a broader range of formant manipulations. Men’s voices are also lower in frequency than are women’s, and as a result men must raise their voices more than women to reach similar high frequency targets. Nevertheless our results indicate that men exceeded the frequency targets reached by women even when raising their voice frequencies to sound small. Indeed we observed extreme maxima in modulations of both *F*0 and VTL, particularly among men. On one hand, this demonstrates an impressive capacity for men to volitionally manipulate their larynges and vocal tracts. On the other hand, it elicits a question about the ecological validity of such extreme modulations, which may be perceived as abnormal.

Our results indicate that speakers modulated *F*0 more than VTL. We also observed asymmetries within each vocal parameter, specifically greater decreases than increases in formants, and greater increases than decreases in *F*0. This latter finding might be explained by nonlinearities in the relationship between vocal fold length and *F*0^32^, and the greater physiological effort required to increase versus decrease vocal fold tension^58^. Indeed baseline *F*0 is closer to the minimum than maximum producible *F*0^12^. As a consequence, sopranos can reach *F*0’s above 1200 Hz, whereas bass singers lower their *F*0 by only a fraction of this magnitude, typically to around 80 Hz^59^.

The demonstrated capacity to volitionally modulate vocal parameters known to be physically related to and perceptually associated with body size can be evolutionarily advantageous, as various indicators of physical size in humans are known to influence a wide range of socioeconomic variables and the mate preferences of both sexes^50^. At the same time, voice modulation is ecologically relevant only if and when it affects listeners. Perceptually, human listeners can discriminate changes in *F*0 or formants of about 5% from a series of vowel sounds^52^, and formant manipulations of 5% are known to affect listeners’ body size estimates^60^. Based on this our results suggest that, on average, men’s formant-based size exaggeration, and both men’s and women’s *F*0-based size reduction, would be perceptually detectable. Studies examining the effectiveness of voice modulation on other types of judgments have produced mixed results^43^,^46^,^47^, but generally suggest that voice modulation may be an effective tool for manipulating listeners’ social judgments of traits such as attractiveness, dominance, and competence. For instance, one recent study found that listeners preferred the voices of men and women whose speech was directed towards attractive individuals, and these preferences were observed for voices recorded in the listener’s own language as well as in a foreign language^47^. In the case of vocally faking a larger body size, and thus a more dominant persona, individuals who are perceived as physically larger due to voice modulation could reap the socioeconomic and reproductive benefits typically linked to these traits across various social contexts including mating, political and marketing contexts. Currently we are conducting playback experiments to test whether vocal modulation can effectively alter listeners’ estimates of body size.

## Methods

### Participants

A total of 167 men and women from Canada (students of McMaster University in Hamilton), Cuba (students of the University of Havana, and staff and students of the Cuban Neuroscience Center in Havana), and Poland (students of the University of Wrocław and the College of Humanities and Economics in Brzeg) took part in the experiment. All participants provided informed consent. Sample characteristics are given in Table 2.

### Procedure

All participants were first recorded speaking the five monophthong vowels /α/, /i/, /ɛ/, /o/, and /u/ (International Phonetic Alphabet) in their natural, baseline voice. Following this, participants were asked to repeat the five vowels while sounding physically small (small condition) and physically large (large condition). These instructions, back translated and given in the native language of the participant, were the only instructions given. Condition order was counter-balanced between participants. Participants then completed a short questionnaire indicating their sex and age. Height was measured using metric tape and weight using an electronic scale. The study was approved by the McMaster Research Ethics Board and methods were carried out in accordance with the approved guidelines.

### Voice recording

All participants were recorded using condenser microphones with a cardioid pick-up pattern at an approximate distance of 5–10 cm (Canada: Sennheiser MKH 800; Cuba: Sennheiser MKH 70; Poland: Audio-M Nova). Audio was digitally encoded with an M-Audio Fast Track interface at a sampling rate of 44.1–96 kHz and 16–24 bit amplitude quantization, and stored onto a computer as PCM WAV files. Recordings from participants at McMaster University and the Cuban Neuroscience Center were conducted in an anechoic sound-controlled booth and recordings at the Universities of Havana and Wrocław were conducted in a quiet room.

### Voice measurement and acoustic analysis

All acoustic measures were performed in Praat^61^. Voice measures were taken from each vowel separately and then averaged across vowels within each vocalizer and condition to obtain mean values. We measured *F*0 using Praat’s autocorrelation algorithm. Following previous work, we set a broad search range of 30–500 Hz for men, and 65–600 Hz for women^41^. We transformed *F*0 measures into equivalent rectangular bandwidth (ERB) units, a quasi-logarithmic scale that controls for the difference between physical and perceived properties of pitch, where 1 ERB is approximately equal to a 40 Hz change at a centre frequency of 120 Hz^62^. The ERB scale correlates strongly with *F*0 in Hz in the range of adult human speech (e.g., *r* = 0.99 in men)^21^.

We measured formants (*F*1–*F*4) using Praat’s Burg Linear Predictive Coding algorithm with the initial settings of maximum formant set to 5500 Hz for women and 5000 Hz for men. Formants were first overlaid on a spectrogram and formant number was manually adjusted until the best visual fit of predicted onto observed formants was obtained. From the mean centre frequencies of *F*1–*F*4 we computed formant spacing, ∆*F*, a measure of the distance among adjacent formants, as well as apparent vocal tract length derived from formant spacing, VTL(∆*F*)^63^. The results of a recent meta-analysis indicate that ∆*F* and VTL(∆*F*) each independently explain more variance in men’s heights and women’s weights than do any other formant measures^17^, and are strongly inversely related (here, *r* = −0.99 within each sex).

Each individual formant is related to ∆*F* by Equation (1):


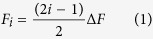


where *i* represents formant position (*F*1–*F*4). Thus, we derived ∆*F* by plotting mean formant frequencies for each individual against the expected increments of formant spacing [(2*i* − 1)/2], where ∆*F* is equal to the slope of the linear regression line with an intercept set to 0^41^,^63^. From this, we estimated the apparent vocal tract length of each individual following equation (2):


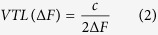


where *c* is 35 000 cm/s, the approximate speed of sound in a uniform tube with one end closed controlling for warmth and dampness (i.e. the vocal tract^12^). From the pooled samples, we confirmed that baseline VTL explained several times more variance in men’s (12%, *r*_S_ = 0.35) and women’s (16%, *r*_S_ =0.40) heights than did baseline *F*0 (2.5% in each sex, *r*_S_ = 0.16; See Supplementary Fig. S1). This pattern of results was similar across samples and agrees with weighted relationships reported at the population level^17^.

### Statistical analysis

We first calculated differences in voice measures between each size condition and baseline, separately for *F*0 and VTL. Positive values indicate increases, and negative values decreases, from baseline. We then ran separate repeated measures ANOVAs for *F*0 and VTL. In each model, the dependent variable was the standardized difference from baseline ([large–baseline]/baseline; [small–baseline]/baseline), controlling for baseline sex differences. Condition (large, small) was included as a within-subject factor, and sex (male, female) and culture (Canada, Cuba, Poland) as between-subject factors. To examine differences in the *magnitude* of voice modulations, we re-ran the models on the absolute standardized difference from baseline in each condition (see Supplementary Information). Significant effects were further examined using planned post-hoc tests. All tests were two-tailed with an alpha of 0.05.

## Additional Information

**How to cite this article**: Pisanski, K. *et al*. Volitional exaggeration of body size through fundamental and formant frequency modulation in humans. *Sci. Rep.* **6**, 34389; doi: 10.1038/srep34389 (2016).

## Supplementary Material

Supplementary Information

Supplementary Audio S1

Supplementary Datset 1

## Figures and Tables

**Table 1:** Means and maxima in VTL (cm) and *F*0 modulation (Hz and ERB) for each sex and condition, given in absolute units and percentage change from baseline.

		Formant Modulation		Voice Pitch Modulation			
		VTL, cm (%)		*F*0, Hz (%)		*F*0, ERB (%)	
		Men	Women	Men	Women	Men	Women
Large Condition	Mean	0.92 (5.3%)	0.54 (3.6%)	−1.03 (0.8%)	−8.57 (4%)	−0.03 (0.8%)	−0.19 (3.1%)
	Maximum	4.00 (23.1%)	3.01 (20.3%)	−43.36 (35.5%)	−75.00 (35.1%)	−1.05 (26.5%)	−1.69 (27.6%)
Small Condition	Mean	−0.51 (3.0%)	−0.20 (1.4%)	32.06 (26.3)	24.80 (11.6%)	0.73 (18.4)	0.48 (7.8%)
	Maximum	−2.79 (16.1%)	−1.65 (11.1%)	345.17 (282.6%)	138.64 (64.8%)	6.30 (159.0%)	2.70 (44.1%)

**Table 2:** Sample characteristics (mean (*s.d*., range)).

Country	Sex	*n*	Age, years	Height, cm	Weight, kg	*F*0, Hz	VTL, cm
Canada	Men	28	19.7 (2.3, 18–30)	177.4 (7.1)	73.4 (11.3)	114.1 (14.6)	17.3 (0.9)
	Women	28	20 (1.4, 18–25)	164.6 (6.8)	60.0 (12.1)	218.2 (22.2)	14.7 (0.6)
Cuba	Men	30	25.9 (2.9, 21–32)	174.6 (6.9)	73.2 (9.6)	131.8 (21.0)	17.4 (0.7)
	Women	25	25.8 (3.2, 21–32)	160.2 (4.6)	58.2 (11.4)	211.7 (18.3)	14.7 (0.6)
Poland	Men	27	28.4 (5.5, 22–42)	181.1 (6.1)	81.3 (11.5)	120.0 (14.7)	17.4 (0.9)
	Women	29	26.4 (6.5, 21–45)	165.1 (5.9)	67.8 (17.1)	211.9 (23.2)	15.2 (0.6)
