# Supplementary material for: Volitional exaggeration of body size through fundamental and formant frequency modulation in humans
Source: Sci Rep. 2016 Sep 30;6:34389. doi: 10.1038/srep34389 (PMC5043380; doi:10.1038/srep34389)
Supplement: Supplementary Information [file srep34389-s1.docx]

**Supplementary Information**

**Volitional exaggeration of body size through fundamental and formant frequency modulation in humans**

Katarzyna Pisanski, Emanuel C. Mora, Annette Pisanski, David Reby, Piotr Sorokowski, Tomasz Frackowiak & David R. Feinberg

The Supplementary Information includes:

- Results – Effects of sex and culture on the magnitude of voice frequency modulations
- Supplementary Figure S1 – Relationships between baseline voice measures and height for each sex.

**Effects of sex and culture on the magnitude of voice modulations**

**Formant or vocal tract length modulation**

A repeated measures ANOVA examining absolute magnitude in VTL modulations showed main effects of condition (*F*_1,111_=14.8.03, *p*<.001,=.12) and sex (*F*_1,111_=10.9, *p*<.001,=.09) and no other effects including of culture. Post-hoc analyses showed that VTL modulations were more extreme in the large than small condition (paired-sample *t*-test: *t*_116_=4.0, *p*<.001). Moreover, the magnitude of men’s VTL modulations exceeded women’s in both the large (one-way ANOVA: *F*_1,132_=4.7, *p*=.033) and small (*F*_1,132_=11.6, *p*=.001) conditions.

**Fundamental frequency modulation**

A repeated measures ANOVA examining absolute magnitude in *F*0 modulations showed main effects of condition (*F*_1,161_=31.3, *p*<.001,=.16), sex (*F*_1,161_=10.6, *p*=.001,=.06) and culture (*F*_2,161_=4.2, *p*=.017,=.05), qualified by interactions between condition and culture (*F*_2,161_=5.7, *p*=.004,=.07) and condition and sex (*F*_1,161_=7.8, *p*=.006,=.05). Absolute modulations in *F*0 were more extreme in the large than small condition (paired-sample *t*-test: *t*_166_=5.4, *p*<.001), and more extreme among men than women in the small condition (one-way ANOVA: *F*_1,166_=9.0, *p*=.003). There were no sex differences in magnitude in the large condition (*F*_1,166_=2.2, *p*=.14). Again *F*0 modulations differed across cultures only in the small condition (*F*_2,166_=4.6, *p*=.011), with more extreme *F*0 modulations among Polish than Canadian participants (Fisher’s LSD: *p*=.003). A difference in magnitude between Cubans and Canadians approached significance (*p*=.061; all other *p*>.27).

**Supplementary Figure S1.** Relationships between baseline voice measures (top panel: vocal tract length, VTL; bottom panel: fundamental frequency*, F*0) and height within each sex. Correlations were computed using non-parametric statistics (spearman’s *rho*) for pooled samples of 85 men and 82 women.
